# Uncultured prokaryotic genomes in the spotlight: An examination of publicly available data from metagenomics and single-cell genomics

**DOI:** 10.1016/j.csbj.2023.09.010

**Published:** 2023-09-12

**Authors:** Koji Arikawa, Masahito Hosokawa

**Affiliations:** a Department of Life Science and Medical Bioscience, Waseda University, 2-2 Wakamatsu-cho, Shinjuku-ku, Tokyo 162-8480, Japan; b bitBiome, Inc., 513 Wasedatsurumaki-cho, Shinjuku-ku, Tokyo 162-0041, Japan; c Research Organization for Nano and Life Innovation, Waseda University, 513 Wasedatsurumaki-cho, Shinjuku-ku, Tokyo 162-0041, Japan; d Institute for Advanced Research of Biosystem Dynamics, Waseda Research Institute for Science and Engineering, 3-4-1 Okubo, Shinjuku-ku, Tokyo 169-8555, Japan; e Computational Bio Big-Data Open Innovation Laboratory, National Institute of Advanced Industrial Science and Technology, 3-4-1 Okubo, Shinjuku-ku, Tokyo 169-8555, Japan

**Keywords:** Metagenomics, Single-cell genomics, Microbiome, Database, Metagenome-assembled genome, Single amplified genome

## Abstract

Owing to the ineffectiveness of traditional culture techniques for the vast majority of microbial species, culture-independent analyses utilizing next-generation sequencing and bioinformatics have become essential for gaining insight into microbial ecology and function. This mini-review focuses on two essential methods for obtaining genetic information from uncultured prokaryotes, metagenomics and single-cell genomics. We analyzed the registration status of uncultured prokaryotic genome data from major public databases and assessed the advantages and limitations of both the methods. Metagenomics generates a significant quantity of sequence data and multiple prokaryotic genomes using straightforward experimental procedures. However, in ecosystems with high microbial diversity, such as soil, most genes are presented as brief, disconnected contigs, and lack association of highly conserved genes and mobile genetic elements with individual species genomes. Although technically more challenging, single-cell genomics offers valuable insights into complex ecosystems by providing strain-resolved genomes, addressing issues in metagenomics. Recent technological advancements, such as long-read sequencing, machine learning algorithms, and in silico protein structure prediction, in combination with vast genomic data, have the potential to overcome the current technical challenges and facilitate a deeper understanding of uncultured microbial ecosystems and microbial dark matter genes and proteins. In light of this, it is imperative that continued innovation in both methods and technologies take place to create high-quality reference genome databases that will support future microbial research and industrial applications.

## 1 Introduction

Microbial research has historically relied on successful isolation and cultivation of microbial species. However, conventional culture techniques are ineffective for more than 99% of microbial species [1], making culture-independent analyses essential for understanding microbial ecology and functions. Breakthroughs in next-generation sequencing (NGS) and bioinformatics technologies have revolutionized the field, allowing culture-independent genome analysis of environmental microbial communities [2], [3]. The vast amount of sequence data now available in the public domain enables meta-analyses to combine data from multiple studies conducted globally. Microbial genetic data is a valuable resource for understanding microbial ecosystems and functions, as well as for identifying industrially relevant enzymes and antibiotics [4], [5].

Metagenomics is a groundbreaking technique for obtaining genomes from uncultured prokaryotes, bypassing the need to culture them [6]. This approach involves directly sequencing the DNA extracted from microbial communities and then assembling the resulting fragmented sequences into contiguous sequences using computer algorithms. The resulting contigs are then grouped into genomic sequence bins for each microbial species. The extensive genetic data provided by metagenomics provide a thorough understanding of the genomic structures and functions of complex microbial communities. Metagenomic applications include exploring the link between obesity and gut microbiota [7], delineating gut microbiota-specific pathways and metabolic modules in patients with inflammatory bowel disease (IBD) [8], and uncovering the unique functions of individual bacteria in specific environments [9].

Single-cell genomics is a method of obtaining uncultured microbial genomes by physically isolating single cells from individual microbial species, amplifying DNA, and sequencing. [10], [11], [12], [13], [14], [15], [16]. Although it requires more complex techniques than metagenomics because it treats single cells and their tiny DNA, recent advances in technology have resulted in a large amount of single-cell genome sequencing data. It is expected that single-cell genomics will provide new insights into genome resolution at the strain level and confirm the findings of metagenomic studies. [5], [17].

In this review, we have analyzed two primary methods for obtaining uncultured prokaryotic genomes: metagenomics and single-cell genomics. We described these techniques, evaluated the current state of uncultured prokaryotic genome data in public databases, and discussed the quality of gene and genome data in the databases based on the origin of the samples and the ecosystem. We then assessed the advantages and limitations of metagenomics and single-cell genomics and explored potential avenues for expanding the data to improve our understanding of uncultured prokaryotic ecosystems and facilitate the industrial application of prokaryotic genes.

## 2 Approaches for obtaining uncultured prokaryotic genes and genomes

### 2.1 Metagenomics

In shotgun metagenomics, DNA fragments extracted from prokaryotic communities are directly sequenced, and sequence reads are then computationally assembled to generate contig sequences as consensus sequences [18], [19]. These contigs, which are composed of sequences from various prokaryotes, are separated into groups to recover the genomes of the individual prokaryotes [20], [21], [22], [23]. This process and recovered genomes are called binning and Metagenome-Assembled Genomes (MAGs), respectively. Various algorithms assign contigs to groups of sequences (bins) based on characteristics, such as GC content, tetranucleotide frequency, and sequence coverage. Because no single binning approach performs well for all metagenomic sequences, bin refinement tools have been developed to consolidate sets of MAGs from different binning predictions [24], [25], [26]. According to our evaluation of the major binning tools [17], CONCOCT [20] and MaxBin 2 [21] tended to put more contigs into the bin, and contamination rates tended to be higher. In contrast, MetaBAT 2 [22] tended to perform conservative binning, and the bin tended to have low contamination and completeness. Bin refinement using DAS_Tool [24] or other tools to extract the reliable MAG from the bin is encouraged.

However, MAGs often contain chimeric sequences from different prokaryotic species [17], [27]. It has been observed that only approximately 7% of MAGs generated from short-read sequencers contain 16S rRNA genes [28], posing challenges in correlating MAGs with 16S rRNA amplicon sequencing. Furthermore, accurately sorting mobile genetic elements, such as plasmids and phages, in MAGs is challenging [29]. Ribosomal protein genes are often not included in MAGs [30]. There are several review articles on metagenomic analysis available; thus, we have not included the details here [31], [32], [33].

### 2.2 Single-cell genomics

In single-cell genomics, individual cells are first isolated from the prokaryotic community using flow cytometric cell sorting or microfluidics [10], [13]. Cell lysis and whole-genome amplification are then performed to obtain sufficient amounts of DNA for sequencing. Single-cell sequence reads are obtained through indexed sequencing, followed by de novo assembly of sequence reads into Single Amplified Genomes (SAGs). Because single-cell genomic sequences are obtained from individual cells, there is no need for contig binning after assembly to produce SAGs, which offers superior genome recovery of rare prokaryotes from complex prokaryotic communities. Single-cell genomics has an excellent recovery of 16S rRNA genes in SAGs and can link prokaryotic host genomes to mobile genetic elements, such as plasmids and prophages [17], [34]. Although SAGs generally exhibit lower genome completeness than MAGs and often include incorrect assemblies by chimeric sequences or external DNA contamination, these problems can be overcome by co-assembly of SAGs and chimera sequence cleaning [11]. While MAGs are population-representative sequences, SAGs are theoretically strain-resolved sequences; therefore, the quality of genome data is not affected by prokaryotic diversity or the presence of similar or dissimilar prokaryotes. Single-cell genomics applications include the analysis of bacteria visible to the naked eye [35], a comprehensive survey of marine bacteria in surface seawater [12], the identification of secondary metabolite producers from marine sponges [36], [37], the assessment of subspecies and intraspecific recombination in environmental bacterial species [38], [39], and the identification of gut bacteria that degrade soluble dietary fiber [13]. There are some technological review articles on single-cell genomics and its future perspectives [40], [41], [42].

### 2.3 Quality control for MAGs and SAGs

A method for assessing the quality of MAGs and SAGs [6] was proposed, which involves classifying them into four categories: finished, high-quality, medium-quality, and low-quality. This classification is based on criteria, such as the degree of genome sequence fragmentation (contig numbers), recovery of rRNA genes, number of tRNA genes, genome completeness, and contamination rate. Genome completeness and contamination are determined using single-copy marker genes with tools like CheckM [43]. High- and medium-quality MAGs or SAGs are usually employed to interpret prokaryotic functions. Open reading frames in the metagenome assembly, MAGs, and SAGs are predicted using prokaryotic gene prediction tools [44], [45], and functional analysis is carried out using COG [46], eggnog [47], and KEGG [48].

## 3 Sequencing data for uncultured prokaryotes in public databases

### 3.1 Raw sequence data

The Short Read Archive (SRA) [49] is a repository for archiving DNA sequence data generated from NGS, which is operated by the International Nucleotide Sequence Database Collaboration (INSDC) [50], including the DNA Data Bank of Japan (DDBJ) [51], European Molecular Biology Laboratory’s European Bioinformatics Institute (EMBL-EBI) [52], and National Center for Biotechnology Information (NCBI) [53]. As of September 2021, the SRA had approximately 25.6 petabases and 17 petabytes of registered DNA bases and file size, respectively [54]. In the two years between September 2021 and May 2023, the number of DNA bases registered in the SRA more than tripled, reaching over 78 petabases.

These data are publicly available, giving all users unrestricted, permanent, and free access [49]. Cloud-based platforms have been developed owing to the requirement for substantial computer resources and bioinformatics expertise for metagenomic analysis [55], [56], [57], [58]. These services provide the analysis, comparison, and storage of metagenomic data, and users can access these datasets via websites, API, or FTP sites.

### 3.2 SRA data collections of metagenomics and single-cell genomics

**Fig. 1:**
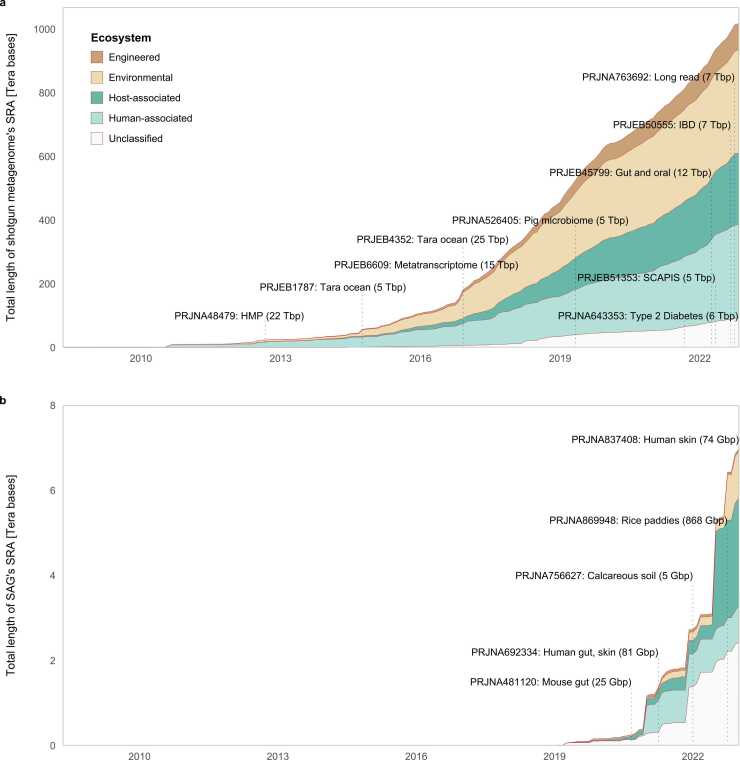
Increase in the SRA data size of metagenomics and single-cell genomics over time. BioProjects with large SRA datasets are shown in boxes. Metadata of the SRA and BioSample information [131] were extracted from SRA_ Accessions.tab and biosample_set.xml.gz, respectively. For the collection of metagenomic data, SRA_Metagenome_Types.tsv provided by PARTIE Github (https://github.com/linsalrob/partie) was used to assign shotgun metagenome sequences. Additional information was extracted from the bioproject.xml and assembly_summary.txt. Single-cell genomic data, including both eukaryotes and prokaryotes, were collected from the SRA if the BioSample package was described as MISAG [6].

Since 2008, metagenomic data have been accumulating in the SRA (Fig. 1a), with the total number of bases exceeding one petabase by 2022. The rate of accumulation is increasing, with several projects registering terabase pair quantities. Prior to 2013, most data were derived from human-associated samples, but since 2014, there has been a significant increase in data from environmental sources. As of 2022, approximately 293 terabases of human-associated samples have been collected, whereas 323 terabases of environmental samples have been collected, which is a reversal of their earlier proportions.

Although the acquisition of single-cell genome sequencing data from environmental microbes was reported in 2007 [59], they were not officially recognized as SAGs in the SRA until 2019 (Fig. 1b). The total number of base pairs exceeded seven terabases by 2022, and the rate of data accumulation is rapidly increasing. While the total sequencing effort for single-cell genomics is generally not as high as that for metagenomics, the total amount of SAG data remains small and equivalent to a single metagenomic project. Most samples have been classified as host-associated or unclassified, with only a small number of registered, environmental samples. With future technological advancements and the increased use of single-cell genomics, it is expected that data from environmental microbes will increase, similar to the growth of metagenomic data.

### 3.3 Web-based analysis platforms for metagenomic data

Shotgun metagenomics requires significant computational resources. According to a report [60], the assembly process requires over 65 GB of memory and 14 h of analysis time depending on the data size and tools used. If a user does not have access to extensive computer resources, they can use analysis platforms such as Integrated Microbial Genome and Microbiomes (IMG/M) [57] or MGnify [58] (previously known as EBI Metagenomics [61]) as alternatives (Table 1).

**Table 1:** Data size of the shotgun metagenome dataset based on public metadata.

Database	Metagenomes	CDSs	Acceptable data	Gene prediction	Functional annotation	Taxonomic annotation	Binning, curation, and assessment	Data accessibility
**IMG/M**	48,866	33,797,010,173	Assembly	• Infernal • Rfam • GeneMarkS-2 • Prodigal • tRNAscan-SE • CRT	• HMMER • COGs • Pfam • TIGRFAM • Cath-FunFam • SuperFamily • SMART • lastal 983 • KEGG Orthology • EC numbers	• lastal • IMG-NR	• MetaBAT • < 3000 bp contigs cutoff • CheckM • GTDB-Tk	• Metadata from website • Data files using API
**MGnify**	33,738	4,821,810,124	Raw reads or Assembly	• Infernal • Rfam • FragGeneScan • Prodigal	• InterProScan • eggNOG-mapper • HMMER • KEGG • Kofam • GO terms • Genome Properties • antiSMASH	• DIAMOND • UniRef90 • MAPseq • SILVA	• MetaBAT 2 • MaxBin 2 • CONCOCT • metaWRAP • < 2500 bp contigs cutoff • CheckM • GUNC • dRep • GTDB-Tk	• Metadata and data files using API • Protein database is sharing via FTP site

IMG/M (https://img.jgi.doe.gov) is a web-based platform for managing and analyzing metagenomic data. It contains annotated DNA and RNA sequences from various microorganisms, including cultured and uncultured bacteria, archaea, eukaryotes, and viruses. Users can upload their DNA assemblies to run the annotation pipeline, which includes predicting protein-coding sequences (CDSs) using GeneMarkS-2 [62] and Prodigal [44], detecting CRISPRs using CRT [63], and predicting RNA features and tRNAs using infernal [64] and tRNAscan-SE [65], respectively. The platform also performs functional annotations for CDSs using various databases including COG, Pfam [66], TIGRFAM [67], Cath-FumFam [68], SuperFamily [69], SMART [70], KEGG Orthology Terms (KO) [71], and Enzyme Commission (EC) numbers derived from KO terms. LAST [72] is used with the UniRef90 reference database [73] for taxonomic annotation of protein-coding genes. The platform incorporates MetaBAT [74] as a metagenome binning tool with a minimum contig cutoff of 3000 bp. After the quality assessment of MAGs by CheckM [43], taxonomic classifications are assigned using GTDB-Tk [75]. The analyzed datasets can be accessed via the website and API. Additionally, users can analyze MAGs in IMGs; however, only some metadata of the registered MAGs are available for bulk downloads (4.5%).

MGnify (https://www.ebi.ac.uk/metagenomics) is an automated pipeline that provides support for both raw reads and assembly of shotgun metagenomic data. The database currently contains 297 different biomes, with over half of the analyses originating from only nine of them: human-associated samples (fecal, oral, digestive system, skin, and unspecified human), marine, soil, mammalian digestive systems, and mixed biome samples. Users can either submit their own data for analysis or browse all analyzed public datasets available in the repository. In the pipeline, CDSs are predicted using Prodigal and FragGeneScan [76]. Non-coding RNAs are identified and annotated using Infernal, tRNAscan-SE, and Rfam [77]. Predicted genes are annotated using InterPro [78], eggNOG [47], and KEGG orthology [79]. Pathway predictions using KEGG and Genome Property [80], gene ontology term assignment, and biosynthetic gene cluster prediction using antiSMASH [81] are also performed. Taxonomic classification is carried out using MAPseq [82] and SILVA [83]. The predicted genes are compared against the UniRef 90 database using DIAMOND [84]. MAGs are recovered by MetaBAT 2 [22], MaxBin 2 [21], and CONCOCT [20] with a 2500 bp minimum contig cutoff and refined using metaWRAP [85]. Chimeric contigs are removed using GUNC [86] and MAGs are dereplicated using dRep [87]. CheckM and GTDB-Tk are performed to evaluate the quality of MAGs and taxonomy assignment, respectively. MGnify offers a website and API to access the dataset, and the protein database can be downloaded in bulk via the FTP site.

**Fig. 2:**
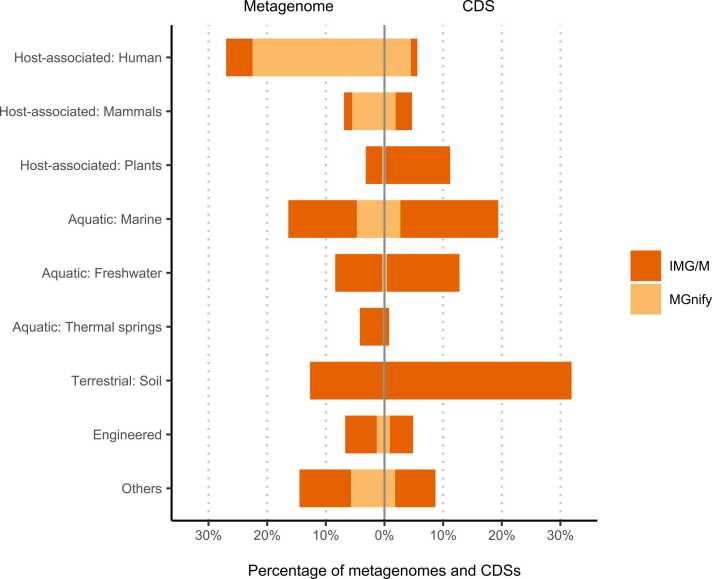
Ecosystem distribution of metagenome assemblies and genes in IMG/M and MGnify. The metagenome (n = 82, 604) and CDSs (n = 38, 618, 820, 297) fractions registered in IMG/M and MGnify were plotted for each representative ecosystem. The color indicates the databases in which the data were registered. The data are based on June 2023.

Table 1 and Fig. 2 show the latest data on shotgun metagenome assemblies in IMG/M and MGnify. IMG/M contains approximately 33 billion CDSs, whereas MGnify has approximately 4.8 billion CDSs. In terms of metagenomic assemblies, IMG/M had the most from environmental samples (n = 35,387, 25 billion CDSs), while MGnify had the most from human-associated samples (n = 18,612, 1.7 billion CDSs).

A combined view of the two major databases (Fig. 2) revealed that human-associated samples comprised 25% of the metagenomic assemblies, followed by marine, soil, freshwater, and mammal-associated samples. Conversely, the soil had the highest proportion of CDSs (30%), followed by marine, freshwater, plant, human, and mammal-associated samples. These results suggest that soil and plant microorganisms, as well as marine and freshwater microorganisms, have more diverse and non-redundant environment-specific genes than those in human-associated samples [88], indicating the importance of expanding genetic resources from environmental prokaryotes for understanding microbial function and industrial applications.

## 4 MAGs and SAGs in public databases

### 4.1 Uncultured prokaryotic genome databases and catalogs

Table 2 shows publicly available databases and data collection for uncultured prokaryotic genomes. The repositories for MAGs and SAGs were obtained from NCBI (https://www.ncbi.nlm.nih.gov/), IMG/M [57], MGnify [58], and the Genome Taxonomy Database (GTDB) (https://gtdb.ecogenomic.org/) [89]. The GTDB is a database that catalogs MAGs and SAGs to establish a standardized microbial taxonomy based on genome phylogeny using a set of single-copy marker proteins based on GTDB-Tk. Specifically, we discuss case studies of SAG, such as WGA-X at the Single Cell Genomics Center, Bigelow Laboratory for Ocean Sciences (ME, US) [12], [15], and SAG-gel (bit-MAP) by bitBiome, Inc. (Tokyo, Japan) [13], [17], [34], which are provided as analysis services with consistent data acquisition and large data sizes.

**Table 2:** MAGs and SAGs in public databases.

Database/catalog or sample (method)	References	MAGs	SAGs
NCBI		130,149	17,270
IMG/M	[57]	232,807	4,830
GTDB	[89]	77,891	831
The Genomes from Earth’s Microbiomes (GEM) catalog	[90]	52,515	
MGnify	[58]	315,252	-
Unified Human Gastrointestinal Genome (UHGG)	[91]	289,232	-
GORG-Tropics (WGA-X)	[12]	-	12,710
Mouse feces (WGA-X)	[15]	-	698
Rice paddy soil (SAG-gel)	[16]	-	4,600
Human skin swab (SAG-gel)	[34], [17]	-	768
Mouse feces (SAG-gel)	[13]	-	346

As of June 2023

**Fig. 3:**
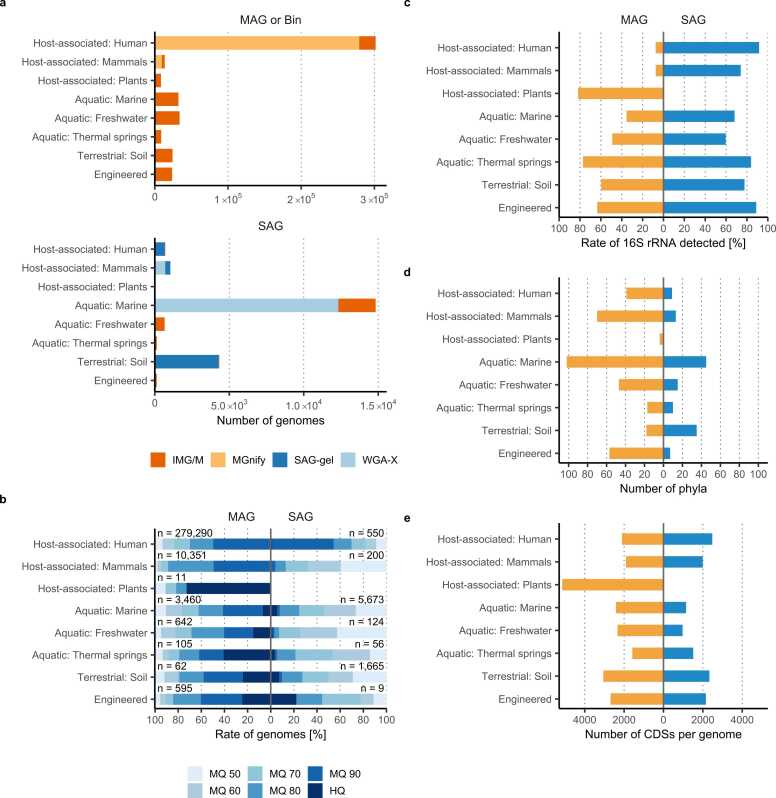
Number and quality of MAGs and SAGs in public databases. Number of MAGs (top) and SAGs (bottom) (a). The MAGs (bins) and SAGs obtained from IMG/M metagenome bins, MGnify genome catalogs, and some BioProjects, including PRJEB33281, PRJDB8805, PRJNA692334, PRJNA837408, and DOI: 10.6084/m9.figshare.c.4454150, were analyzed for each ecosystem. Genome completeness of MAGs (left) and SAGs (right) (b), presence rate of the 16S rRNA gene (c), number of phyla (d), and number of CDSs per genome (e) were plotted. Only the medium- and high-quality genomes are shown in (b)–(e). The data are based on June 2023.

With regard to the microbial habitats classified as ecosystems in Fig. 3a, in the IMG/M, approximately 37% of MAGs were derived from aquatic environments, followed by approximately 10% from soil environments. IMG/M includes the Genomes from Earth’s Microbiomes (GEM) catalog (https://portal.nersc.gov/GEM/), which was constructed from 10,450 metagenomes sampled from diverse microbial habitats and geographic locations [90]. Approximately 70% of MAGs in Earth’s microbiomes are derived from the human gut or marine environments. In IMG/M, CDS in the metagenome assembly were more abundant in soil than in marine environments (Fig. 2), but the number of MAGs was reversed, indicating the difficulty of constructing MAGs from soil metagenome assemblies. MGnify has the largest number of MAGs (304,283), with approximately 90% of these MAGs derived from the human gut, which is referred to as the Unified Human Gastrointestinal Genome (UHGG) catalogue [91].

Although SAG datasets are one order of magnitude smaller than MAG datasets, the two datasets derived from the ocean [12] and soil environments [16] are larger than other SAG collections, with some projects acquiring thousands to tens of thousands of SAGs. To date, no cross-habitat microbial or large cohort-based genome collection projects, such as the GEM catalog or UHGG, have been undertaken in studies collecting SAGs.

### 4.2 Qualities of MAGs and SAGs

The statistical data for MAGs and SAGs are shown in Fig. 3. Genome completeness tended to be higher in MAGs (average of 85.3%) than in SAGs (Fig. 3b). MAGs are often selected and registered for bins of medium or higher quality in most projects or catalogs [89], [90], [91], [92], [93], [94]. However, even in human and mammalian samples with a large number of registered MAGs, few MAGs can be classified as high quality. This is due to the low recovery rate of the 16S rRNA genes, which will be discussed later.

The majority of SAGs had a completeness of less than 90%, with an average completeness of 71.0% (Fig. 3b). Conventional single-cell genomics, especially based on flow cytometric cell sorting and the conventional microtube WGA reaction, has very low genome completeness and high contamination rates due to amplification bias, chimera occurrence, and contamination [95], [96], [97]. The number of successfully amplified single cells can vary greatly from sample to sample, and sample-specific experimental optimization is necessary to obtain the best results. Freshwater and marine samples have been reported to have the highest percentage of successfully amplified genomes (up to 40%), whereas soil samples tend to have lower success rates (less than 10%) [95]. However, these shortcomings in genome amplification for single-cell genomics have been addressed by WGA-X, an improved whole-genome amplification enzyme [12], [15], or microfluidic droplets, which use droplets or gel capsules for cell isolation and genome amplification [10], [13], [14], [96], [98], [99]. SAGs derived from human skin using SAG-gel [100] were comparable in genome completeness (85.7%) to those of human-associated MAGs. Depending on the sample type, SAGs with genome completeness similar to that of MAGs were obtained (Fig. 3b). In addition, single-cell genomics has the unique feature of integrating multiple SAGs derived from cells of the same species or strain to improve genome quality [11], [14], [101]. Furthermore, by integrating SAGs with MAGs obtained from the same sample, uncultured prokaryotic genomes with improved accuracy, covering the lack of information in MAG, can be obtained [17], [102], [103], [104].

A major challenge associated with MAGs is the lack of 16S rRNA gene sequences. The presence of skewed species abundance, high 16S rRNA sequence similarity, and dependence on short-read sequencing make it difficult to assemble individual prokaryote-specific 16S rRNA genes from complex prokaryotic communities [105]. It has been reported that only 7% of MAGs have 16S rRNA gene sequences in more than 270,000 human gut MAGs, showing over 95% completeness and less than 5% contamination [28]. As shown in Fig. 3c, this lack of 16S rRNA genes was consistently observed in MAGs, with yields below 10%, particularly in MAGs from human- and mammalian-associated metagenomes. This can be attributed to a significant species bias in symbiotic bacteria and the high similarity of 16S rRNA genes between symbiotic bacteria. Conversely, 16S rRNA gene yields in SAGs were significantly higher than those in MAGs, regardless of ecosystem. The low 16S rRNA gene recovery in MAGs hinders the linking of taxonomy to functional genomic information. To address this methodological gap, the active use of SAGs and the curation of reliable MAGs [89], [90], [91], [93], [94] is crucial in microbiome research.

### 4.3 Genetic diversities in MAGs and SAGs

There are fewer than 20,000 prokaryotic species with valid published names, representing less than 0.2% of the estimated prokaryotic species diversity [106]. Most prokaryotes are not available as pure cultures and, therefore, cannot be named according to the rules and recommendations of the International Code of Nomenclature of Prokaryotes (ICNP). A code called SeqCode [107] was proposed to effectively publish prokaryotic names based on isolated genomes, MAGs, and SAGs. SeqCode uses genome sequence data as a common currency for typing cultivated and uncultivated microbes, and follows rules similar to those of ICNP for priority.

Prokaryotic genomes, including isolates, MAGs, and SAGs, are typically classified using taxonomic classification tools like GTDB-Tk [75]. The classification system consists of seven major ranks: species, genus, family, order, class, phylum, and domain. While many MAGs were obtained from human and mammalian samples (Fig. 3a), the number of phyla was relatively small (Fig. 3d). This suggests that the genomes of limited microbial lineages are frequently encountered in human- or mammalian-associated samples, and that the genomes of diverse microbial lineages are more likely to be found in environmental samples. For human-related microbiome samples, a thorough understanding of microdiversity is essential. Although not included in this review, various databases have been developed for gut bacteria and other organisms [91], [108], [109], [110], [111], [112], [113], [114]. Single-cell genomics should be utilized to acquire genomes of known species at the strain level from human-associated samples [17], [34], [100] and to identify the genomes of novel species in environmental samples where metagenomic binning is not feasible.

### 4.4 Exploring prokaryotic genes from public genomes

In terms of the number of CDSs per genome (Fig. 3e), SAG had fewer CDSs per genome (1469 CDSs) than MAG (2108 CDSs), which is consistent with the lower genome completeness of SAG compared to MAGs. SAGs obtained using SAG-gel had a similar number of CDSs (2470, 2329, and 1875 CDSs) to MAGs in humans [17], [100], soil [16], and mammal-associated samples [13], respectively. In mammals, SAGs obtained using WGA-X also had 2025 CDSs [15].

**Fig. 4:**
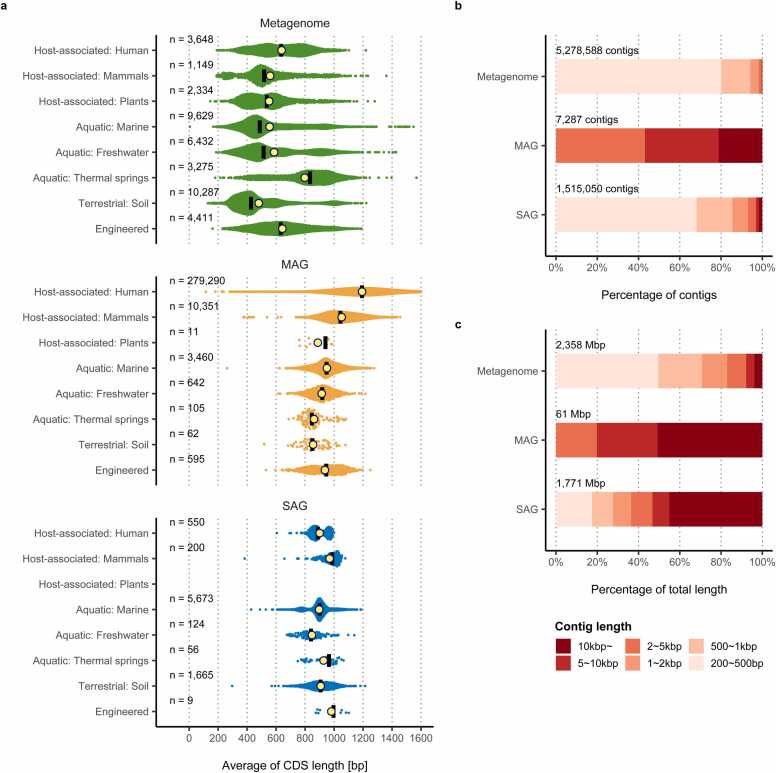
Average length of genes in metagenome assemblies, MAGs, and SAGs. The average length of the CDSs per sample was plotted according to ecosystem classification (a). The dataset is same as Fig. 3b. Metagenome assemblies, MAGs, and SAGs are colored green, orange, and blue, respectively. The mean and median values are indicated by yellow circles and bars, respectively. Comparisons of the presence of short contigs are presented in (b) and (c). The number of contigs and total length of soil metagenomes, MAGs, and SAGs were plotted against their abundance ratios separately for each contig length stage (b) (c). Metagenomes and MAGs from BioProject PRJNA375197 and SAGs from BioProject PRJNA869948 were used.

It is essential to identify full-length genes when exploring useful genes such as enzymes. To evaluate this, the average length of CDSs per metagenome assembly and MAG in IMG/M or SAG in WGA-X or SAG-gel was calculated (Fig. 4a). The MAGs and SAGs analyzed here correspond to either high- or medium-quality genomes. The average length of CDSs from MAGs and SAGs was consistently 900–1000 bp across different ecosystems. However, the average length of CDSs from metagenome assemblies was significantly shorter than those of MAGs and SAGs. In soils, the average lengths of CDSs in MAGs and SAGs were 856 bp and 908 bp, respectively, whereas the average length of CDSs in the metagenome assemblies was 481 bp. To analyze this in detail, we examined the contig numbers and sizes of soil metagenome assembly under 200 bp cutoff and revealed that 94.2% of contigs were less than 1 kbp, and these short contigs accounted for 70.8% of the total length (Fig. 4b, c). In contrast, in the SAG, short contigs (<1 kbp) accounted for 85.5% of the contigs, but their total length was only 27.7%, and long contigs (>10 kbp) accounted for over 40% of the total size. In the process of MAG construction, cutoffs of short contigs of less than 3000 bp are generally used. Thus, there were no short contigs in soil MAGs (Fig. 4b, c). The total number of contigs assigned to MAG was quite small, at 0.14% of the total metagenome assemblies, consistent with a previous report [88]. The length of the predicted CDSs in metagenomic assemblies and MAGs depends mainly on the length of the contigs. Although it is desirable to perform gene searches with reference to microbial lineages, most of the contigs on soil metagenomes are short and are discarded during the binning process; therefore, a limited number of CDSs must be used when using MAG as the search source. Therefore, there is a great possibility that soil metagenomes and MAGs may not provide adequate information as a gene discovery resource [88]. Regarding gene prediction, it is also important to consider that partial genes originating from the edges of contigs. Approximately 85% of the genes in the MGnify protein database were partial.

Most genes are specific to a single habitat, and the technical challenge is to efficiently recover rare, habitat-specific, and region-specific genes [88]. Prospects include using MAG and SAG to develop searches for specific enzymes from target prokaryotic species with characteristics such as lack of pathogenicity and industrial accessibility. This will also require improved gene function prediction techniques, including protein structure prediction and search [115], [116], [117], [118], [119], to identify unknown genes.

## 5 Summary and outlook

The number of uncultured prokaryotic genomes is growing rapidly, and some are publicly available in databases. Metagenomics and single-cell genomics help us identify species and their proteins in prokaryotes from various environments, such as soil, ocean, and even inside the human body. However, little is known about uncultured prokaryotic genes and proteins, beyond their nucleic acid or primary amino acid sequences, making uncultured microbial proteins the 'dark matter' of the protein universe. We are now in an era where this dark matter can be elucidated by adapting state-of-the-art protein structure prediction methods to vast uncultured prokaryotic genome data [117], [119], [120]. Advanced analysis of uncultured prokaryotic genes will help solve evolutionary history mysteries, discover proteins that can cure diseases, clean up the environment, and produce clean energy.

This mini-review provided an overview of uncultured prokaryotic genes and genomes in public databases and evaluated the quality of data available in each ecosystem. This highlighted that while shotgun metagenomics provided a large number of genes, fragmented contigs in ecosystems made it difficult to obtain full-length genes. It is challenging to construct multiple species-resolved MAGs from complex prokaryotic populations because most contigs are unassigned and discarded in the binning process [88]. The use of long-read sequencing technologies, such as PacBio [121], [122], [123] and Oxford Nanopore Technologies [124], [125], [126], [127], can help overcome these issues. Future developments in binning algorithms that leverage machine learning [23], [128] and Hi-C metagenomics [121], [129], [130] may help address these challenges.

However, the quality of SAGs is not affected by specific ecosystems, and SAG can provide complementary information to MAGs, such as 16S rRNA genes and mobile genetic elements. Single-cell genomics is a highly effective method for obtaining unknown species genomes and strain-resolved genomes, especially in environmental samples containing diverse prokaryotes. We suggest using single-cell genomics as a valuable strategy for gaining insight into and conducting a comprehensive analysis of complex ecosystems without the need for complex computing processes, such as metagenomic binning. However, challenges for single-cell genomics include expanding the number of SAGs that can be acquired in a sequencing run, reducing costs, and simplifying the method. We anticipate that continued advancements in this field will lead to the development of an integrated approach between metagenomics and single-cell genomics, resulting in a high-quality prokaryotic genome database.

## Funding

This work was partially supported by MEXT/JSPS KAKENHI 21H01733 and JST FOREST JPMJFR210F.

## CRediT authorship contribution statement

**Koji Arikawa**: Conceptualization, Validation, Software, Formal analysis, Data curation, Visualization, Investigation, Writing- Original draft preparation. **Masahito Hosokawa:** Conceptualization, Supervision, Writing- Reviewing and Editing, Funding acquisition.

## Declaration of Competing Interest

K.A. is employed at bitBiome, Inc, which provides single‐cell genomics services using the SAG‐gel workflow as bit‐MAP. M.H. is a founder and shareholder of bitBiome, Inc.
